# Effect of five year storage on total phenolic content and antioxidant capacity of almond (*Amygdalus communisL.*) hull and shell from different genotypes

**Published:** 2015

**Authors:** Khadijeh Sadat Moosavi Dolatabadi, Gholamreza Dehghan, Siavash Hosseini, Ali Jahanban Esfahlan

**Affiliations:** 1 *Department of Biology, Faculty of Science, Urmia University, Urmia, Iran, I. R. Iran*; 2 *Department of Biology, Faculty of Natural Science, University of Tabriz, Tabriz, Iran, I. R. Iran*; 3 *Biotechnology Research Centre, Tabriz University of Medical Sciences, Tabriz 51664-14766, Iran*; 4 *Department of Pharmaceutical Biotechnology, Faculty of Pharmacy, Tabriz University of Medical Sciences, Tabriz 51664-14766, Iran*

**Keywords:** *Almond*, *Hull*, *Shell*, *Genotype*, *Radicalscavenging*, *Storage*

## Abstract

**Objectives**: Almond (*Prunus amygdalus*) hull and shell are agricultural by-products that are a source of phenolic compounds.The processing of almond produce shell and hull, accounts for more than 50% by dry weight of the almond fruits. Recently, more studies have focused on the influence of storage conditions and postharvest handling on the nutritional quality of fruits, especially the antioxidant phenolics. In this study, influence of long-term storage (five years) on the total phenolic and antioxidant capacity of almond hull and shell from different genotypes was evaluated.

**Materials and Methods**: The fruits of subjected genotypes were collected and their hull and shell were separated. They were dried and reduced to fine powder. This powder stored at room temperature for five years. The total phenolic content (TPC) and bioactivities (antioxidant potential: DPPH and ABTS radical scavenging and reducing power) of extracts were evaluated using spectrophotometric methods.

**Results: **It was found that TPC content and bioactivity levels in the stored almond hull and shell were different, compared to the hulls and shells which were evaluated in 2007. S_1_-4 genotype had the highest TPC and reducing power in its hull and shell.Low correlation coefficient was observed between phenolic content and the DPPH radical scavenging percentage in hull and shell extract.

**Conclusions**: For the first time, results of this investigation showed that storage can influence the antioxidant and antiradical potential of almond hull and shell.

## Introduction

In the past decade, a growing interest in biology and medicine has been focused on identifying substances and food components that can protect against oxidative stress. Natural antioxidants have a wide range of biological activities, including inhibition of ROS generation, direct or indirect scavenging of free radicals, and alteration of intracellular redox potential (Dehghan et al., 2007 ▶). Generally, abroad studies identified that a wide variety of phenolic acids and flavonoids present in fruits and nuts. Those compounds are primarily conjugated with carbohydrates or other polyols via O-glycosidic or ester bonds (Barreira et al., 2008 ▶). In fact, phenolic compounds prevent lipid oxidation by eliminating free radicals, chelating metals, activating antioxidant enzymes, reducing tocopherol radicals, and inhibiting enzymes that create oxidation reactions (Heim et al., 2002 ▶). Flavonoids are a large group of polyphenolic natural compounds that are favorable to human health due to their variety of biological and pharmacological properties including cardiovascular protection, anticancer, antiulcer, anti-allergic, antioxidant, antiviral, and anti-inflammatory potentials (Dehghan and Khoshkam, 2011 ▶). 

The anticancer activity of nuts has also been demonstrated in experimental animals. This beneficial effect is due to their lipid profile, arginine, fiber, and vitamin E contents as well as to other compounds with antioxidant properties such as polyphenols (Monagas et al., 2007 ▶).

Almond, scientifically known as *Prunusdulcis*, belongs to the *Rosaceae *family, produced on a global basis (Barreira et al., 2008 ▶; Wijeratne et al., 2006 ▶). Iran is optimally situated for growing almonds and is the center of origin for almonds (Gorttapeh et al., 2006 ▶). Almond fruit consists of 4 portions: the kernel, the brown skin of the meat or the seed coat the middle shell, and the outer green shell cover or almond hull. The nutritional importance of almond fruit is related to its kernel. Almond shell and hull were used as livestock feed and fuel source (Jahanban et al., 2009, 2009 ▶). It is well-known that fruits and nuts contain a wide variety of phenolic acids and flavonoids that are predominantly conjugated with sugars or other polyols via O-glycosidic or ester bonds and its consumption has been associated with reduced risk of chronic diseases (Pellegrini et al., 2006 ▶). This genus is reported to have interesting biological properties such as sedative, anti-inflammatory, anti-hyperlipidemic, anti-tumoral, and antioxidant activities (Donovan et al., 1998 ▶; 1999; Sang et al., 2002a ▶). 

In clinical trials, almond (*Prunusdulcis*) consumption increased the intake of vitamin E (VE) and monounsaturated fats and also reduced plasma low-density lipoprotein (LDL), lipoprotein(a), and insulin resistance (Jaceldo-Siegl K, et al. 2004 ▶).Moreover, actions consistent with observational data showed the decrease in risk of cardiovascular disease (Lovejoy JC, et al., 2002 ▶).

Almond hull and shell are produced with processing of almonds in amounts for more than 50% by dry weight of the fruit. Extracts of whole almond seed, brown skin, shell, and hull possess potent antioxidant and radical scavenging capacities. These activities are due to the presence of flavonoids and other phenolic compounds in nuts (Moure et al., 2007 ▶). Various phenolic compounds have been detected in almond by-products (Wijeratne, 2006 ▶). Almond hull contain triterpenoids, sterols (Takeoka and Dao, 2003 ▶), lactones (Sang et al., 2002a ▶), and phenolics (Sang et al., 2002b ▶). Takeoka and Dao (2003) ▶ analyzed the methanolic extract of almond hull by using reverse phase HPLC with diode array detection. The extract contained 5-O-caffeoylquinic acid (chlorogenic acid), 4-O-caffeoylquinic acid (cryptochlorogenic acid), and 3-O-caffeoylquinic acid (neochlorogenic acid). Almond shell is highly lignified (30–38% of the dry weight) (Martinez et al., 1995 ▶) and the antioxidant potential of depolymerized lignin fractions produced after mild acid hydrolysis of ligno-cellulosics has been reported (Gonzalez et al., 2004 ▶). Phenolic compounds naturally exist in a phenolic acid form as hydroxybenzoic acid or hydroxycinnamic acid derivatives and are usually covalently bound to insoluble polymers. This limits the activity of bound phenolic compounds as natural anti-oxidative agents and there have been attempts to liberate bound phenolic acids (Choi et al., 2011 ▶). The content of phytochemical substances is influenced by various factors such as ripening time, genotype, cultivation techniques, and climatic conditions that occur during the pre-harvest period but also the operations carried out during the post-harvest storage are very important (Lee and Kader, 2000 ▶).

Universally, underlined different factors (genotype, cultivation techniques, climatic conditions that occur during the pre-harvest period, post-harvest storage conditions, and processing) which may affect the chemical composition of plant foods and they may have an important role in determining the phenolic composition and the bioactivity of these compounds. Some studies have shown that during storage, the content of the total and individual phenolic compounds remain relatively permanent, while other studies have detected minor fluctuations in phenolic concentrations (Awad and Jager, 2003 ▶; Imeh and Khokhar, 2002 ▶; Lata, 2008 ▶). Storage can influence the quality indices and nutritional content of fresh fruit. During post-harvest storage of agricultural crops, significant changes in antioxidant status can occur (Tavarini et al., 2008 ▶). The aim of this study was to evaluate influence of long-term storage (five years) on the total phenolic and flavonoid contents and antioxidant capacity of almond hull and shell from different genotypes. 

## Materials and Methods

**Sample preparation**

Almond fruits of 18 A. *communis* L. genotypes were collected from different locations (Qooshchi, Qalgachi, QovarchinQale, Najaf Abad, Jamal Abad, Khariz, and Esfahlan) of West and East Azerbayjan provinces of Iran at the end of September 2007. The hull and shell of those almonds were separated. Then, they were dried and reduced to fine powder. This powder was stored at room temperature for five years (2007-2012).

**Preparation of extracts**

For extraction of the antioxidant compounds, one gram of each powder sample was extracted with 20 ml of pure methanol in a soxhlet apparatus at 60 °C for 30 min (Wijeratne et al., 2006 ▶). The yield extracts were filtered through filter paper and stored at 4 °C.

**Determination of Total phenolic contents**

The total phenolic contents were determined with Folin-Ciocalteu Reagent (FCR) according to the method of Singleton and Rossi (1965) ▶ by some modifications. Briefly, 0.5 ml of each phenolic extract was mixed with 2 ml of 7.5% sodium carbonate and then the mixture was allowed to stand at room temperature for 2 min. After addition of 2.5 ml ten-fold Folin-Ciocalteu reagent, the mixture was incubated in the dark room for 30 min. The absorbance was measured at 720 nm using a spectrophotometer. The analysis was performed in triplicate and the concentration of phenolic compounds was expressed as mg of gallic acid equivalents (GAE) per gram of extract.

**Reducing power**

The reducing power of the hull and shell methanolic extract was determined according to the method of Oyaizu (1986) ▶. Hull and shell methanolic extract (1 ml), phosphate buffer (1 ml, 0.2 M, pH 6.6), and potassium ferricyanide (1.0 ml, 10 mg/ml) were mixed together and incubated at 50 °C for 20 min. Trichloroacetic acid (1.0 ml, 100 mg/ml) was added to the mixture and centrifuged at 13,400 g for 5 min. The supernatant (1.0 ml) was mixed with distilled water (1.0 ml) and ferric chloride (0.1 ml, 1.0 mg/ml), and then the absorbance was measured at 700 nm. 

**DPPH free radical scavenging activity**

The DPPH radical scavenging activity was determined as described by Brand-Williamsetet al. (1995) with some modifications. The stock solution was prepared by dissolving 0.004 g DPPH with 100 ml methanol. Fifty µl of the hull and shell extracts was added to 950 µl of 2,2-diphenyl-1-picrylhydrazyl (DPPH) solution (0.1 mM in methanol) and the reaction mixture incubated at room temperature for 10 min. Then, the absorbance of this solution was determined at 517 nm using a spectrophotometer. The radical scavenging activity (RSA) was calculated as a percentage of DPPH discoloration using the following equation:

RSA% = (A _blank_ – A _sample_) /A _blank_ ×100

**ABTS** ^+^ **assay**

For ABTS^+^ radical scavenging activity assay, the procedure followed the method of Pennycooke et al. (2005) ▶ with some modifications. Briefly, 54.2 mg of ABTS^+^ powder was dissolved in 10 ml of phosphate buffer (5 mM, pH 7.0) and mixed with 1 g of MnO_2_ and incubated in room temperature within 30 min for the generation of green colored ABTS^+^. The prepared solution was centrifuged for 5 min and after filtration the filtrate was diluted with phosphate buffer until the absorbance of the solution equals with 0.70 ± 0.01 in 723 nm. Hull and shell extracts (50 µl) were mixed with 950 µl of ABTS^+^ solution and incubated for 10 min at room temperature. The decrease of absorbance was monitored at 734 nm after 10 min. The percentage of radical inhibition activity was calculated according to the following equation:

RSA% = (A_o_– A_f_) /A_o_ × 100

Where A_o_ is the absorbance of the un-inhibited radical cation and A_f_ is the absorbance measured 10 min after addition of the samples.

**Statistical analysis**

All of the assays were obtained from triplicate measurements and results are expressed as means ± standard deviations. The Statistical Package for Social Sciences (SPSS, version 20 for windows) was used to analyze the data. The means were compared using Duncan’s multiple range (DMRT) test at *p*< 0.05 following analysis of variance (ANOVA).

## Results

**Total phenolic content**

**Figure 1:**
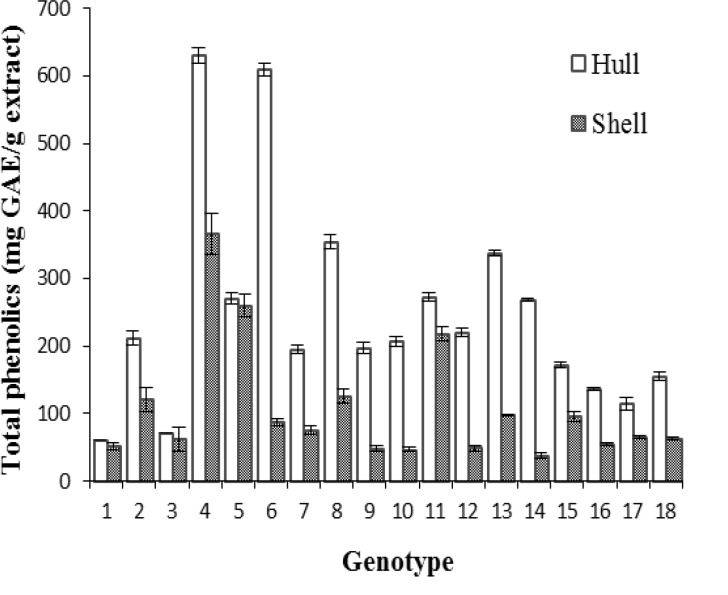
Phenolic content in 18 genotypes of A. communis L. stored hulls and shells: 1: S1-1, 2: S1-2, 3: S1-3, 4: S1-4, 5: S2-5, 6: S2-6, 7: S3-7, 8: S3-8, 9: S4-1, 10: S4-2, 11: S4-3, 12: S4-4, 13: S4-5, 14: S4-6, 15: J1-1, 16: J1-2, 17: N1-1, 18: K1-1. Mean of three replicates with S.E, p< 0.05.

The amount of total phenolics in stored almond hull and shell of 18 genotypes is shown in Figure 1. 

**Reducing power**

**Figure 2:**
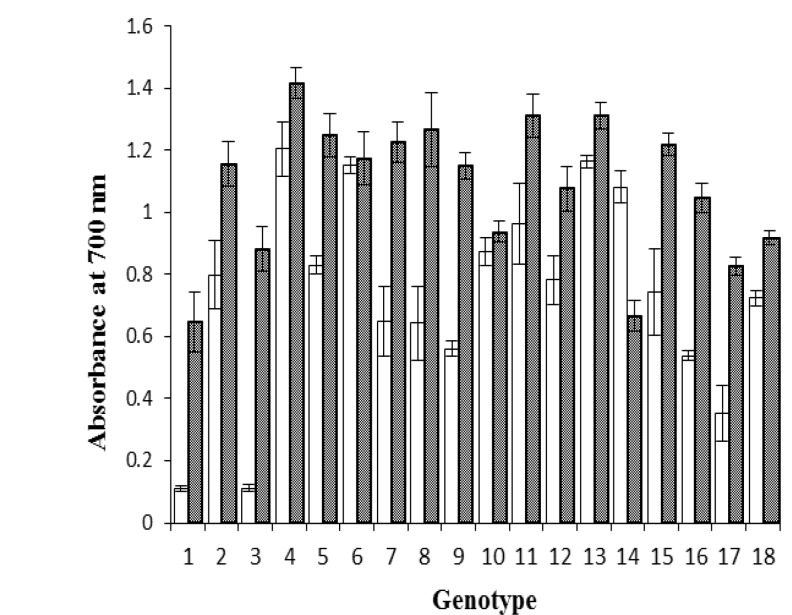
Reducing power in 18 genotyps of A. communis L. stored hulls and shells: 1: S1-1, 2: S1-2, 3: S1-3, 4: S1-4, 5: S2-5, 6: S2-6, 7: S3-7, 8: S3-8, 9: S4-1, 10: S4-2, 11: S4-3, 12: S4-4, 13: S4-5, 14: S4-6, 15: J1-1, 16: J1-2, 17: N1-1, 18: K1-1. The hull extracts diluted 50 fold. (Mean ± S.E, n = 3), p < 0.05

The reducing power of stored almond hull and shell extracts of 18 genotypes were significantly different (Figure 2).

**DPPH radical scavenging activity**

**Figure 3:**
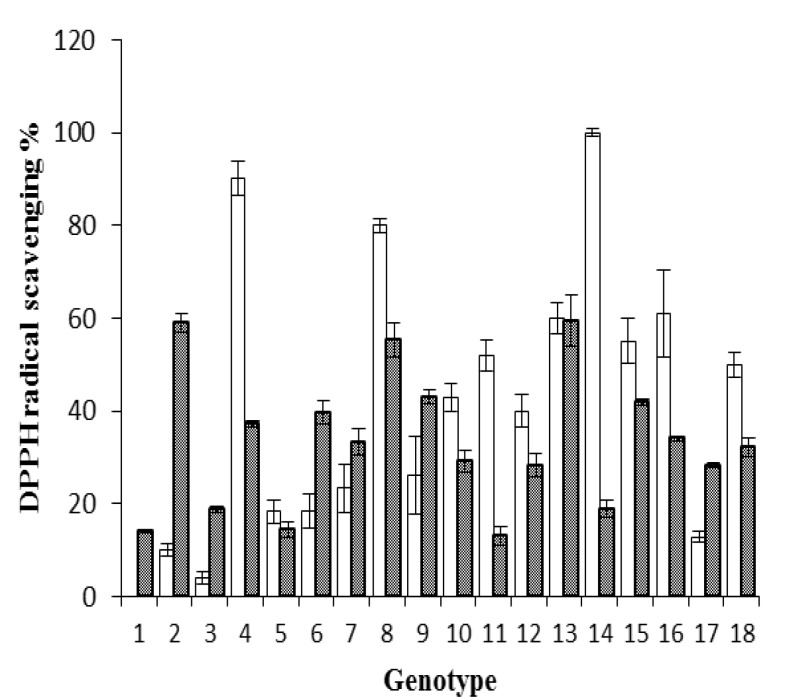
DPPH radical scavenging activity in 18 genotypes of A. communis L. stored hulls and shells: 1: S1-1, 2: S1-2, 3: S1-3,4: S1-4, 5: S2-5, 6: S2-6, 7: S3-7, 8: S3-8, 9: S4-1, 10: S4-2, 11: S4-3, 12: S4-4, 13: S4-5, 14: S4-6, 15: J1-1, 16: J1-2, 17: N1-1, 18: K1-1. The hull extracts were diluted 100 fold. (Mean ± S.E, n = 3), p< 0.05

The radical scavenging capacity of the extracts was determined by transformation of DPPH radical into its reduced form of DPPHº-H (Figure 3).

**Figure 4:**
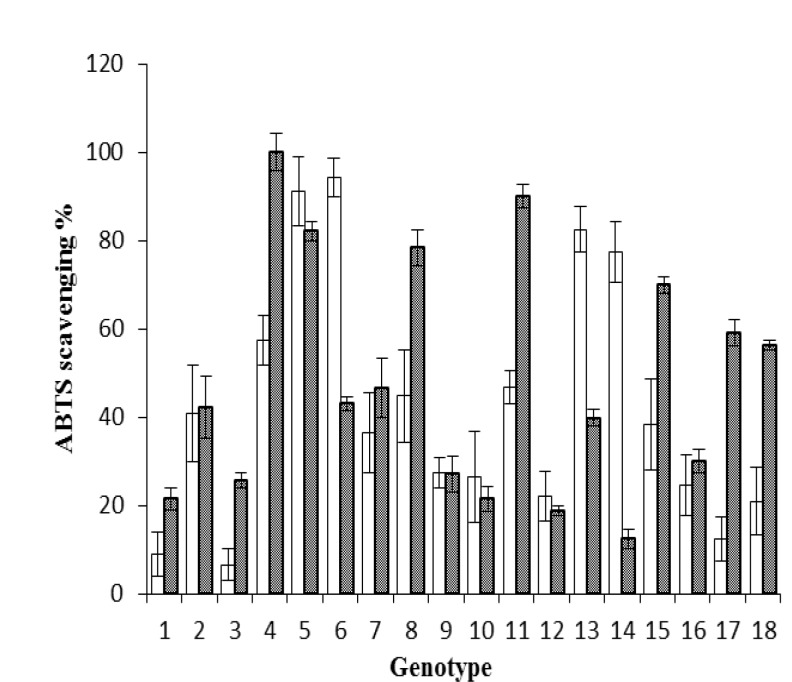
ABTS^+^ radical scavenging activity in 18 genotypes of A. communis L. stored hulls and shells: 1: S1-1, 2: S1-2, 3: S1-3, 4: S1-4, 5: S2-5, 6: S2-6, 7: S3-7, 8: S3-8, 9: S4-1, 10: S4-2, 11: S4-3, 12: S4-4, 13: S4-5, 14: S4-6, 15: J1-1, 16: J1-2, 17: N1-1, 18: K1-1. The hull extracts diluted 60 fold. (Mean ± S.E, n = 3), p < 0.05

**ABTS** ^+^ **radical scavenging activity**

For evaluating the anti-radical potential of the stored almond hull and shell extracts, we used the ABTS^+^ assay (Figure 4).

## Discussion

**Total phenolic content**

There were significant differences (p< 0.05) among phenolic contents in the hull and shell of different genotypes. The content of total phenolic compounds of the stored almond hull was higher than that of the stored shell in each genotype.

Among the stored hull and shell of different genotypes, the maximum total phenolic content was 630 ± 12 and 366 ± 30 mg GAE/g extract for S1-4 genotypes in its hull and shell, respectively. Whereas in the study of Jahanban et al. (2009) ▶ the means of total phenolic contents for almond hull and shell extracts were equal to 78.2 ± 3.41 and 38.0 ± 3.30 mg GAE/g extract. Therefore, no decrease was observed in the amount of total phenolics in almond hull and shell during storage for five years. 

These results are supported by those of Choi et al.(Choi et al., 2011 ▶) who reported that the TPC values (free phenolic acids and soluble phenolic acid esters) in the long-term stored Chenpi was higher than the regular stored Chenpi. These results are also in agreement with those results reported by Hoang, Golding, and Wilkes(Hoang et al., 2011 ▶) that the level of phenolics significantly increased twofold in the flesh of apple during cold storage. In an another study,Tavarini et al.(Tavarini et al., 2008 ▶) found that a significant rise was occurred in phenol content in kiwifruit after the 6 months storage at 0^ º^C which further increased after a week at ambient temperature.Generally, phenol content may either increase or decrease in fruits and vegetables depending on the storage conditions. 

Previous studies showed that almond polyphenolics include flavonolsisorhamnetin, kaempferol, and quercetin, as well as the flavanone naringein (Frison-Norrie S, & Sporns P. 2002 ▶; Sang et al., 2002a ▶; Milbury PE, et al., 2006 ▶) each of which possesses anti-inflammatory, vasodilatory, and antioxidant activities. 

The increase of TPC value with the long storage period have resulted from enzymatic hydrolysis or from biodegradation of previously un-extractable bound phenolic compounds over the extended storage period (Choi et al., 2011 ▶).

**Reducing power**

The absorbance values of the hull extract at different genotypes were obtained to be higher than (p< 0.05) that of shell extract. This result was according to the result of Jahanban et al. (2009) ▶ five years earlier.

The mean absorbance values for stored almond hull extracts, which were diluted 50 fold, was 0.74 ± 0.06. For stored almond shell extracts, the mean of reducing power was obtained 1.1 ± 0.06. However, in the study of jahanban et al. (2009) ▶, the mean reducing power of almond hull and shell extracts obtained 0.519 and 0.228, respectively. 

Almonds are a good dietary source of vitamin E, sterols, and flavonoids, each of which has been suggested to play a role in the promotion of health. In particular, increased consumption of flavonoids has been associated with an anti-obesity effect and reduced risk of stroke, cardiovascular disease, and some forms of cancer (Hertog, M. G. et al., 1993 ▶). These results indicated that the reducing power of almond hull and shell was not decreased during long storage period.

**DPPH radical scavenging activity**

Significant differences (p< 0.05) were observed among hull and shell in each genotype (Figure 3). The extracts of almond hulls were diluted 100 fold, therefore, stored hulls extract showed to bea potent scavenger of DPPH radical. These results are in agreement with previous studies that showed total antioxidant activity was steady or increased during storage (Awad and Jager,2003 ▶; Lata, 2008 ▶). Choi et al. (Choi et al., 2011 ▶) indicated that DPPH radical activity for free phenolic acid increased with extended storage period, however, no big changes were associated with the extended storage period in soluble phenolic acid esters. The DPPH scavenging activity was reduced in insoluble bound phenolic acids fraction whereas TPC values for this fraction remained permanent during long storage period.

Hoang et al. (Hoang et al., 2011 ▶) also reported that the total antioxidant activity in both the peel and flesh tissue of apple increased significantly during storage by 40% and 70%, respectively. Low correlation coefficients were obtained between total phenolic content and radical scavenging activity in hull and shell extracts (R^2^=0.091 and R^2^=0.169, respectively). However,in a previous study,Jahanban et al. (2009) ▶ observed a high correlation between radical scavenging activity and phenolic content. These results indicate that phenolic compounds in almond hull and shell of various genotypes were altered by storage for five years.

**ABTS** ^+^ **radical scavenging activity**

Significant difference (p< 0.05) was obtained between radical scavenging activities of different genotypes (see Figure 4). The extracts of almond hull were diluted 60 fold. Thus, similar to DPPH method, ABTS^+^ radical scavenging activity of the hull extracts was higher than (p< 0.05) that of the shell extracts. 

In this study phytochemical contents and anti-oxidizing activities measured as DPPH and ABTS^+^ radical-scavenging activities were evaluated in stored almond hull and shell from different genotypes. The TPC, reducing power, and antioxidant capacity generally increased with storage (five years). Our findings indicate that the long storage period did not decrease the phytochemical contents that possess almost equivalent bioactivity. Moreover, long storage period requires no additional process to maintain this activity level.
